# Metagenomic Insights into *Candidatus* Scalindua in a Long-term Cultivated Marine Anammox Consortium: The Important Role of Tetrahydrofolate-mediated Carbon Fixation

**DOI:** 10.1264/jsme2.ME25007

**Published:** 2025-06-17

**Authors:** Thelwadanage Nadisha Tharangani Kumari Nawarathna, Naoki Fujii, Kohei Yamamoto, Kyohei Kuroda, Takashi Narihiro, Noriatsu Ozaki, Akiyoshi Ohashi, Tomonori Kindaichi

**Affiliations:** 1 Department of Civil and Environmental Engineering, Graduate School of Advanced Science and Engineering, Hiroshima University, 1–4–1, Kagamiyama, Higashi-Hiroshima, Hiroshima 739–8527, Japan; 2 Bioproduction Research Institute, National Institute of Advanced Industrial Science and Technology (AIST), 2–17–2–1 Tsukisamu-Higashi, Toyohira-ku, Hokkaido 062–8517, Japan

**Keywords:** *Candidatus* Scalindua, anammox, Wood–Ljungdahl pathway, tetrahydrofolate (THF), folate biopterin transporter (FBT)

## Abstract

Marine anammox bacteria have been an exciting research area in recent years due to their high effectiveness in treating ammonia-containing saline wastewater. However, their direct implementation in the wastewater industry faces challenges due to slow growth, difficulty obtaining pure cultures, and their tendency to exist as part of an anammox consortium, interacting symbiotically with other bacteria. In the present study, 91 draft genome metagenome-assembled genomes (MAGs) from a long-term-operated reactor were recovered to clarify detailed symbiotic interactions within an anammox consortium. One marine anammox bacterial MAG, identified as *Candidatus* Scalindua, was successfully recovered and was abundant within the sampled microbial community. A comprehensive metabolic pathway ana­lysis revealed that *Ca.* Scalindua exhibited the complete anammox pathway and the Wood–Ljungdahl pathway for carbon fixation. The folate biosynthesis pathway in *Ca.* Scalindua was incomplete, lacking dihydrofolate reductase, a key enzyme for tetrahydrofolate (THF) production. The folate biopterin transporter, essential for transporting folate-related metabolites among coexisting bacteria, was identified exclusively in *Ca.* Scalindua. In addition, the impact of exogenously supplied THF on microbial activity and carbon uptake rates was investigated in batch experiments using ^14^C-labeled bicarbonate. The results obtained revealed that 2‍ ‍mg L^–1^ of exogenous THF resulted in a 43% increase in the carbon uptake rate, while anammox activity remained unaffected. The present results suggest that THF is a key intermediate for carbon fixation in *Ca.* Scalindua and may be essential for their growth.

Since its discovery in wastewater sludge in the early 1990s, anaerobic ammonium oxidation (anammox) has seen significant improvements and attracted considerable research interest as a nitrogen removal process (Kuenen, 2008; Yang *et al.*, 2022). Anammox bacteria, classified into the phylum *Planctomycetota* within the Planctomycetes-Verrucomicrobia-Chlamydiae (PVC) group, exhibit chemolithotrophic behavior, enabling the direct conversion of ammonium directly into nitrogen and the production of energy-rich hydrazine under anaerobic conditions using nitrite as an electron acceptor and ammonia as an electron donor (Oshiki *et al.*, 2022). Compared with conventional nitrogen removal methods, anammox technology is suitable for various wastewater treatment processes. This is due to the properties of anammox bacteria, including their low energy requirements, high nitrogen removal capacity, high salt tolerance, endurance of high temperature variations, and ability to survive in very low-oxygen environments with minimum external carbon resources (Kindaichi *et al.*, 2011; Gonzalez-Martinez *et al.*, 2018; Trinh *et al.*, 2021). Anammox bacteria are found in diverse ecological environments, from marine to freshwater and soil (Dalsgaard *et al.*, 2005; Kartal *et al.*, 2006; Zhu *et al.*, 2011). *Candidatus* Scalindua (Kuypers *et al.*, 2003; van de Vossenberg *et al.*, 2013) has been recognized as the predominant anammox bacteria in marine environments, accounting for 30–50% of nitrogen removal therein (Tan *et al.*, 2023).

Metagenomic ana­lyses have recently been conducted to examine the genomic diversity of freshwater anammox bacteria. The findings obtained revealed that inorganic-based freshwater anammox bacteria have a symbiotic relationship with other organic-based bacteria, including *Proteobacteria*, *Actinomycetota*, *Chloroflexota*, *Bacteroidota*, and the superphylum *Patescibacteria* (Hosokawa *et al.*, 2021). These symbiotic microorganisms interact, exchanging nutrients, enzymes, substrates, DNA, and metabolites via a cross-feeding phenomenon (Lang and Beatty, 2000; Smith *et al.*, 2019). Additionally, anammox bacteria generate numerous extracellular polymeric substances, which provide organics for heterotrophic bacteria in anammox consortia (Ji *et al.*, 2021). Ongoing research is investigating secondary biosynthesis, cofactors, and vitamins alongside the main metabolic processes necessary for executing vital metabolic processes (Zhao *et al.*, 2018). Information on the metabolic processes and symbiotic relationships of marine anammox bacteria with other coexisting microbial communities is limited in current scientific literature.

Six natural carbon fixation pathways have been identified within bacterial metabolic processes. These include the Calvin–Benson–Bassham, reductive tricarboxylic acid, 3-hydroxy propionate bi, 4-hydroxybutyarate/3-hydroxy propionate, and dicarboxylate/4-hydroxybutyrate cycles; and the acetyl-CoA/Wood–Ljungdahl and reductive glycine pathways (Garritano *et al.*, 2022). The Wood–Ljungdahl pathway is an energy-efficient autotrophic carbon fixation process that operates under anaerobic conditions. Discovered in the early 1980s in Gram-positive (acetogenic bacteria) and methane-producing archaea (methanogenic archaea), this pathway is now recognized in various phylogenetic phyla, including some *Proteobacteria*, *Planctomycetota*, *Actinomycetota*, and *Spirochaetota*, which often perform folate metabolism (Ragsdale, 2008). Anammox bacteria are chemolithoautotrophic, utilizing organic compounds as electron donors and CO_2_ as their primary carbon source to synthesize the cellular biomass (Berg *et al.*, 2010; Martin, 2020). Among the *Planctomycetota* group, only anammox bacteria exhibit a complete Wood–Ljungdahl carbon fixation pathway (Hügler and Sievert, 2011). In this pathway, two CO_2_ molecules from two parallel processes join the pathway to form acetyl-CoA. One CO_2_ molecule is reduced to a methyl group using a tetrahydropterin coenzyme, while the other is reduced to carbon monoxide (CO). The methyl group and CO combine with nickel radicals and release coenzyme A to form an acetyl-CoA molecule (Cohen *et al.*, 2020).

Secondary metabolites, cofactors, and vitamins are needed to complete autotrophic carbon fixation. Previous studies identified metabolites involved in molybdenum cofactor (MOCO) and folate biosynthesis, which are crucial for the Wood–Ljungdahl carbon fixation pathway (Ya *et al.*, 2022). In addition, tetrahydrofolate (THF), a secondary folate biosynthesis metabolite, is used in the Wood–Ljungdahl carbon fixation pathway as a building block for methyl compounds, including 10-formyl tetrahydrofolate and 5,10-methyl tetrahydrofolate. However, most folate pathways in anammox bacteria appear to be incomplete (Zhao *et al.*, 2018; Zhou *et al.*, 2020). Therefore, other coexisting bacteria are hypothesized to provide THF metabolites to anammox bacteria via cross-feeding interactions in the anammox consortium. The mechanisms by which THF metabolites are transported between anammox and symbiotic bacteria and the impact of THF metabolites on the activity and growth of anammox bacteria currently remain unclear.

Therefore, the present study investigated the metabolic pathways of marine anammox and coexisting bacteria in a marine anammox consortium cultivated stably under laboratory conditions for more than 15 years using a metagenomic approach. Furthermore, the impact of THF supplementation on anammox activity and the Wood–Ljungdahl carbon fixation pathway was investigated through batch experiments using ^14^C-labeled bicarbonate.

## Materials and Methods

### Reactor operation and chemical ana­lysis

Biomass samples were collected from a lab-scale up-flow column reactor (volume of 300‍ ‍mL) fitted with a non-woven fabric sheet and operated for more than 15 years, as previously reported by Kindaichi *et al.* (2011). The up-flow column reactor was continuously supplied with a synthetic marine medium, slightly modified from that described by Kindaichi *et al.* (2011). It contained 30‍ ‍g‍ ‍L^–1^ SEALIFE (Marine Tech); (NH_4_)_2_SO_4_ (23.5–33.5‍ ‍mg-N L^–1^), NaNO_2_ (35–43‍ ‍mg-N L^–1^), and KHCO_3_ (1,000‍ ‍mg L^–1^) as nutrient sources; along with MgSO_4_·7H_2_O (300‍ ‍mg L^–1^), KH_2_PO_4_ (27‍ ‍mg L^–1^), CaCl_2_·2H_2_O (180‍ ‍mg L^–1^), and 1‍ ‍mL each of trace element solutions I and II, as described by van de Graaf *et al.* (1996). Before adding the nutrient solution, the substrate was degassed for 1‍ ‍h with N_2_ gas to remove dissolved oxygen (DO). pH was adjusted using H_2_SO_4_ to control the influent pH at 7.0–7.5. The concentration of NH_4_^+^ was measured using Nessler’s method with a UV-visible spectrophotometer (DR 2800; Hach Company) (Kambara *et al.*, 2022). To measure NO_2_^–^ and NO_3_^–^, samples were prepared by filtering through a membrane with a pore size of 0.20‍ ‍μm (Advance Tec). NO_2_^–^ and NO_3_^–^ concentrations then assessed using ion chromatography (HPLC 10 Avp; Shimadzu).

### Sample collection, sequencing, and assembly

Three separate biomass samples were collected from the lab-scale up-flow *Ca.* Scalindua reactor fed with synthetic marine medium in April 2019 (day 3,876), August 2019 (day 4,044), and June 2020 (day 4,346) (Fig. S1). These samples were collected at different time points to capture potential variations in the microbial community over time. Fujii *et al.* (2022) provided a detailed description of the DNA extraction, sequencing, and assembly methodology adopted in the present study. Briefly, DNA was extracted using a Fast DNA Spin kit for soil (MP Biomedicals) according to the manufacturer’s instructions and subsequently purified using Agencount AMPure XP magnetic beads (Beckman Coulter). DNA extracted from the three separate samples was used to generate three separate libraries for Illumina short-read sequencing and PacBio long-read. Illumina sequencing libraries were prepared using a TruSeq DNA PCR-free kit, with paired-end sequencing for short reads (2×151 bp) on a HiSeqX Illumina Platform (Illumina). PacBio sequencing libraries (long reads) were prepared using a SMRT bell Express Template prep kit (PacBio) on a PacBio sequel II system. Circular consensus sequence (CCS) reads were generated from sequel data with a Phred quality score >20 (Q20, 99%). Raw paired-end reads from HiSeqX were trimmed using Trimmomatic version 0.39 (SLIDING WINDOW; 4:30, MINLEN: 100) (Bolger *et al.*, 2014). Three different sets of trimmed HiSeqX and PacBio CCS reads were assembled using SPAdes version 3.15.4 with K numbers of 21, 31, 41, 51, 61, 71, 81, 91, 101, 111, and 127 (Bankevich *et al.*, 2012) to produce coverage files for metagenomic binning. Moreover, contigs exceeding 1,500‍ ‍bp were separated using SeqKit version 2.2.0 (Shen *et al.*, 2016). Minimap2 release 2.17-r941 (Li, 2018) was used to align the whole genome and obtain mapping information.

### Binning, quality assessment, and annotation

Metagenomic binning was performed through a multidimensional coverage approach with several tools, including MetaBAT2 version 2.15 (Kang *et al.*, 2019), MaxBin2 version 2.2.7 (Wu *et al.*, 2016), MyCC (MyCC_2017.ova, Lin and Liao, 2016), and Vamb version 3.0.2 (Nissen *et al.*, 2021). Optimized binning results were obtained using DasTool version 1.1.5 (Sieber *et al.*, 2018). The completeness and contamination of each metagenome-assembled genome (MAG) were evaluated using CheckM version 1.2.0 (Parks *et al.*, 2015). Relative abundance at the genus level was estimated using CoverM version 0.6.1 for each of the three samples, and the average value is shown in Table 1.

### Genome annotation and functional gene identification

Genome annotation was conducted using PROKKA version 1.13 (Seemann, 2014) and BlastKOALA (Kanehisa *et al.*, 2016) from the Kyoto Encyclopedia of Genes and Genomes (KEGG) database. A heat map was constructed using a KEGG decoder (Graham *et al.*, 2018) to visualize the percentage of gene possession related to each gene set. The genome tree was constructed using a combination of GTDB-Tk version 2.2.6 (R207) and IQ-TREE version 2.2.2.3. Conserved marker genes were identified using “gtdbtk identify” as the default parameter. Phylogenetic trees were constructed using an auto-optimized surrogate model (LG+F+I+R10) in IQ-TREE (B1000) (Minh *et al.*, 2020). Furthermore, functional genes related to nitrogen metabolism, folate metabolism, and the Wood–Ljungdahl carbon fixation pathway were thoroughly identified using PROKKA and BlastKOALA. The predicted functional genes were taxonomically confirmed using BLAST P against the non-redundant protein sequences (nr) database. Outputs were further filtered based on identity, with more than 80% identified for nitrogen metabolism and 50% for folate metabolism and carbon fixation in the Wood–Ljungdahl pathway.

### Batch experiments

Batch experiments were performed to investigate the effects of exogenously added THF on anammox activity and carbon uptake, as summarized in Table S1. A 0.5-mL biomass sample (0.35‍ ‍mg protein vial^–1^) and 4‍ ‍mL of the synthetic marine medium were transferred into 10-mL vials. Regarding THF samples, 0.5‍ ‍mL of THF was added, while for control samples, 0.5‍ ‍mL of the synthetic marine medium (excluding [NH_4_]_2_SO_4_, NaNO_2_, and KHCO_3_) was used. The vials were degassed with N_2_ gas at 0.04‍ ‍MPa for 3‍ ‍min to remove DO. Samples were then pressurized with N_2_ gas at 0.04‍ ‍MPa for 10‍ ‍s to prevent air contamination before adding ^14^C. ^14^C-labeled bicarbonate (Sodium [^14^C] bicarbonate, specific activity 2.146 GBq mmol^–1^) (Revvity) was added to vials at a final concentration of 37 kBq vial^–1^. Thereafter, vials were sealed with a butyl rubber plug and aluminum seal and then incubated at 28°C for 24 h. An additional experiment involved pasteurizing the anammox biomass at 70°C for 15‍ ‍min to test for adsorption phenomena. Biomass concentrations were measured as protein using a TaKaRa BCA Protein Assay kit (Takara Bio). After the incubation, all biomass samples were fixed by adding 2.5‍ ‍mL of 12% paraformaldehyde to the 5-mL culture, yielding a final concentration of 4% paraformaldehyde. The uptake of ^14^C was confirmed by liquid scintillation counting. The ^14^C content was calculated from 1‍ ‍mL of the total culture sample (^14^C-containing medium plus biomass) and 1‍ ‍mL of the washed ^14^C-containing biomass sample (obtained by resuspending in 1‍ ‍mL of pure water after centrifugation at 10,000×*g* for 8‍ ‍min following three washes with phosphate-buffered saline, pH=7.4). Each 1-mL sample was mixed with 9‍ ‍mL of scintillation cocktail (Clear-sol I; Nacalai Tesque), and radioactivity was measured using an LSC-6100 liquid scintillation counter (ALOKA). Since the variation between the two samples was unequal, Welch’s *t*-test was performed using R version 4.3.1 (R CoreTeam, 2023).

### Nucleotide sequence accession number

Metagenomic sequence data were deposited in the DDBJ database under the DDBJ/EMBL/GenBank accession number PRJDB17738.

## Results and Discussion

### Genome reconstruction and general genome features

In total, 0.77 billion reads were generated collectively from the sequencing process (Tables S2 and S3). After removing low-quality bases by trimming, 0.39 billion high-quality reads were generated from HiSeq X short-read sequencing. A total of 9,333 contigs over 1,500 bp were obtained from these sequence reads and used in the multiple-dimensional coverage binning approach, generating 91 MAGs. The 25 most abundant MAGs (23.3%) were then listed based on a criterion of <6% contamination and >70% completeness (Table 1). The total abundance of the 25 MAGs accounted for 76.3%. Fig. S2 shows a phylogenetic tree of the 25 recovered genome MAGs based on protein sequences. These 25 MAGs were associated with *Planctomycetota* (6 MAGs), *Proteobacteria* (8 MAGs), *Myxococcota* (2 MAGs), *Bacteroidota* (2 MAGs), *Actinomycetota* (3 MAGs), *Chloroflexota* (2 MAGs), *Spirochaetota* (1 MAG), and *Verrucomicrobiota* (1 MAG). The most abundant MAG (23.3%) was closely related to *Ca.* Scalindua (GCA_004282745.1). The unclassified *Planctomycetota* genome, with the second highest abundance (19.4%), was closely related to the uncultured, genus-level lineage PNC21 in *Phycisphaerales*. The third most abundant MAG belonged to the phylum *Proteobacteria*. The abundance of the phyla *Chloroflexota*,* Verrucomicrobiota*, and *Bacteroidota*, which showed close symbiosis with freshwater and marine anammox bacteria in previous studies (Ali *et al.*, 2020; Oshiki *et al.*, 2022; Oba *et al.*, 2024), was extremely low in the present study.

### Metabolic ana­lysis

**Fig. 1.**
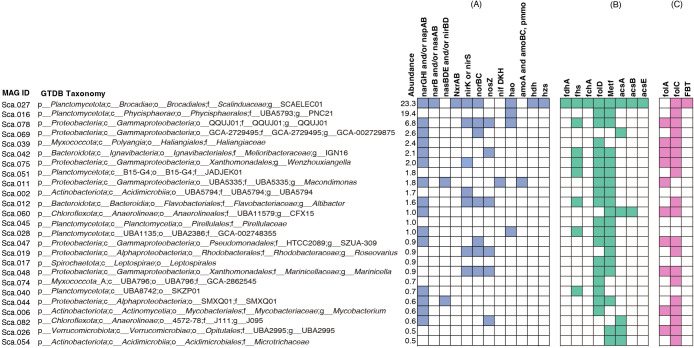
A heatmap showing the presence and absence of functional genes associated with nitrogen metabolism, carbon fixation, and folate metabolism. Gene presence was confirmed using a BLAST P reference dataset, with nitrogen metabolism-related genes considered to be present at >80% identity and carbon fixation and folate metabolism genes at >50% identity. (A) Nitrogen metabolism-related functional genes. narGHI and napAB, nitrate reductase; narB, ferredoxin-nitrate reductase; nasAB, assimilatory nitrate reductase catalytic subunit; nasBDE, nitrite reductase (NADCP)H large subunit; nirBD, nitrite reductase (NADH) larger subunit; nxrAB, nitrite oxidation; nirK/nirS, nitrite reduction; norBC, nitric oxide reduction; nosZ, nitrous oxide reduction; nifDKH, nitrogenase molybdenum-iron protein alpha chain; hao, hydroxylamine oxidoreductase; amoABC, Pmmo, ammonia oxidation; hdh, hydrazine dehydrogenase; hzs, hydrazine synthase subunit. (B) Wood–Lundahl carbon fixation pathway-related functional genes. fdhA, formate dehydrogenase alpha subunit; fhs, formate THF ligase; fchA, methyl-THF cyclohydrolase; fold, methylene-THF dehydrogenase (NADP^+^); Metf, methylene-THF reductase (NADH); acsA, carbon monoxide dehydrogenase; acsB, acetyl-CoA synthase; and acsE, 5-methyl-THF corrinoid/iron-sulfur protein methyltransferase. (C) Folate biosynthesis-related functional genes. folA, Dihydrofolate reductase; folC, folylpolyglutamate synthase; and FBT, folate-biopterin transporter. After the ana­lysis, nitrogen metabolism-related functional genes were sorted at >80%, and carbon fixation and folate metabolism-related functional genes were sorted at >50%.

The most highlighted MAG in this study, *Ca.* Scalindua (Sca.027 MAG), clearly showed high completeness of the TCA cycle, gluconeogenesis, and glycolysis metabolism (Fig. S3). Moreover, concerning nitrogen metabolism, the Sca.027 MAG showed all related genes for anammox metabolism, including hydrazine dehydrogenase (*hdh*), with 100% identity with *Ca.* Scalindua sp. (Accession Number KAA3603839.1), and hydrazine synthase (*hzs*), with 92.4% identity with *Ca.* Scalindua rubra (Accession Number ODQ59245.1) (Fig. 1A). Sca.016, the second most abundant genome affiliated with the UBA5793 family in the phylum *Planctomycetota*, was predicted to possess a complete assimilatory nitrate reduction pathway, including *narB* and *nasB* (Fig. 1A).

Fig. 1B shows the key enzymes of the Wood–Ljungdahl carbon fixation pathways for all 25 MAGs, including the formate dehydrogenase alpha subunit (fdh), formate THF ligase (fhs), methyl THF cyclohydrolase (fchA), methyl THF dehydrogenase (folD), and methyl THF reductase (metf) for the methyl branch and carbon monoxide dihydrofolate (acsA), acetyl-CoA synthase (acsB), and 5-methyl-THF corrinoid/iron-sulfur protein methyltransferase (acsE) for the carbonyl branch. However, only the genome of the *Ca.* Scalindua-related MAG, Sca.027, clearly showed a complete Wood–Ljungdahl carbon fixation pathway, encoding all specific key enzymes with high identity (BLAST P). We confirmed that the Sca.027 MAG lacked the complete set of genes required for the five other carbon fixation pathways based on BlastKOALA results. Since *Ca.* Scalindua is recognized as an anaerobic autotrophic bacterium, it primarily utilizes the Wood–Ljungdahl pathway for carbon fixation (Ragsdale and Pierce, 2008). Conversely, all other MAGs exhibit an incomplete carbon fixation pathway characterized by heterotrophic bacterial behavior.

Formate dehydrogenases, metabolite products of MOCO biosynthesis, and THF from the complete folate biosynthesis pathway are involved in the Wood–Ljungdahl pathway of anammox bacteria (Zhuang *et al.*, 2014). The present study primarily focused on investigating marine anammox folate biosynthesis, the effects of THF metabolites on the anammox carbon fixation pathway, and marine anammox bacteria growth potential. Notably, marine anammox bacteria (MAG Sca.027) exhibited an incomplete folate biosynthesis pathway (Fig. 1C), lacking the dihydrofolate reductase (folA) enzyme essential for THF production. Dihydrofolate reductase is one of the critical enzymes for converting dihydrofolate to THF and is involved in purine and thymidylate synthesis (Lin and Gerson, 2014).

**Fig. 2.**
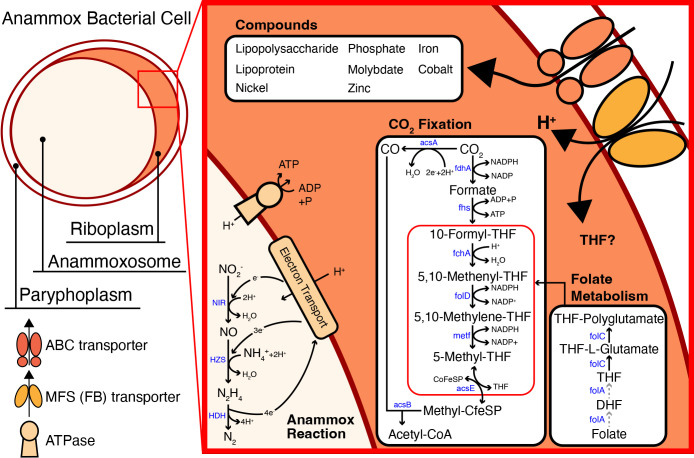
Overview of predicted metabolic pathways. The pathway containing secondary metabolism is related to the Wood–Ljungdahl carbon fixation pathway in marine anammox bacteria. The black solid line indicates a complete pathway, and the gray dashed line represents an incomplete pathway in the folate biosynthesis pathway.

The Sca.078 (*Proteobacteria*), Sca.042 (*Bacteroidota*), Sca.060 (*Chloroflexota*), Sca.026 (*Verrucomicrobiota*), and Sca.006 (*Actinobacteriota*) MAGs exhibited a complete folate biosynthesis pathway (*i.e.*, possessing folA and folC) with high sequence identities based on a BLAST P ana­lysis. Previous studies proposed that cross-feeding transfers may be crucial for facilitating the exchange of deficient enzymes or secondary metabolites between anammox bacteria and other symbiotic microorganisms (Zhou *et al.*, 2020). Precisely defining the cross-feeding mechanism and identifying suitable transporters that facilitate the transfer of THF metabolites between species is necessary. Therefore, we conducted a comprehensive ana­lysis to examine the presence of folate transporters within MAGs. The folate biopterin transporter (FBT) in the Sca.027 MAG (*Ca.* Scalindua) was identified using PROKKA and confirmed via a BLAST P ana­lysis as an MFS transporter with 100% identity (KAA3592863.1) (Fig. 1C). FBT transporters are proton-coupled symporters that move folates and biopterin across membranes, primarily in bacteria and other lower organisms (Fig. 2) (Eudes *et al.*, 2010). However, further research is needed to identify more specific roles for and the mechanisms of these transporters. Furthermore, we compared the recovered *Ca.* Scalindua MAG (Sca.027) with 30 other *Ca.* Scalindua MAGs (Table S4). Our ana­lysis revealed that all *Ca.* Scalindua MAGs lacked folA, the genome encoding dihydrofolate reductase responsible for metabolizing folate to DHF and THF. It is possible that anammox bacteria obtain the necessary secondary metabolites to complete carbon fixation through mechanisms such as uptake from dead cells, cell fusion, cell-cell interactions, or bacterial membrane vesicles (Venturi and Bez, 2021; Toyofuku *et al.*, 2023). Furthermore, a key observation in the ana­lysis of Wood–Ljungdahl metabolism is that the THF generated during the conversion from 5-methyl-THF to methyl-CfeSP undergoes a re-reaction with folate, thereby completing the Wood–Ljungdahl carbon fixation pathway. This catalytic process is facilitated by the 5-methyl-THF corrinoid/iron-sulfur protein methyltransferase (acsE) enzyme, present only in MAG Sca.027.

### The impact of exogenous THF on carbon fixation

**Fig. 3.**
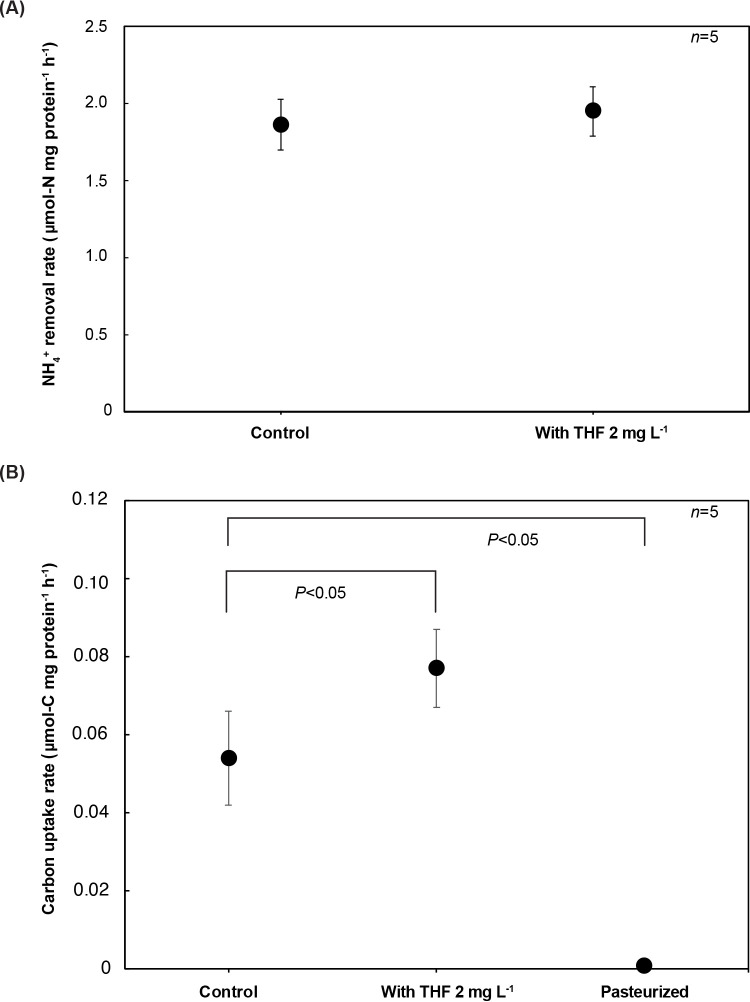
Effects of tetrahydrofolate (THF) on anammox activity (A) and carbon uptake (B).

The impact of exogenous THF on laboratory-cultivated marine anammox bacterial consortia was investigated through batch experiments. The effects of THF on carbon fixation were assessed by quantifying the carbon uptake rate. Additionally, consequential effects on anammox bacterial activity were exami­ned. The average ammonia removal rates in control and THF-supplemented samples were 1.86±0.17 and 1.95±0.16‍ ‍μmol-N mg protein^–1^ h^–1^ (Fig. 3A), and carbon uptake rates were 0.054±0.02 and 0.077±0.01‍ ‍μmol-C mg protein^–1^ h^–1^, respectively (Fig. 3B). Pasteurized samples showed a significantly low carbon uptake rate due to the inactivation of enzymes and microbial activity.

These results demonstrated that the carbon uptake rate was significantly higher in THF-supplemented samples than in control samples, while no significant differences were observed in ammonia removal rates between these two conditions. It is important to note that biomass yield efficiency was calculated as 0.029‍ ‍μmol-C μmol-N^–1^ in the control, which was consistent with previous findings (Awata *et al.*, 2013). On the other hand, biomass yield efficiency with the THF addition (2‍ ‍mg L^–1^) was 0.040‍ ‍μmol-C μmol-N^–1^, which showed a 38% increase. Further confirmation that exogenously introduced components enhance biomass yield in *Ca.* Scalindua is needed (Awata *et al.*, 2015; Mojiri *et al.*, 2018). Exogenous THF may exert complex effects on the Wood–Ljungdahl carbon fixation pathway. In cases where *Ca.* Scalindua is deficient in THF, supplementation with THF may simulate the methyl branch of the carbon fixation pathway by providing the necessary cofactors (Fig. 2). This may lead to increased acetyl-CoA production and an enhanced carbon uptake capacity. Furthermore, FBT facilitates the translocation of THF across the biological membrane. However, since THF is also produced as a byproduct of the Wood–Ljungdahl carbon fixation pathway, exogenous THF may not always be required for effective fixation.

## Conclusion

The present study performed a comprehensive metagenomic ana­lysis and evaluated the effects of exogenous THF supplementation in a marine anammox consortium that has been cultivated under controlled laboratory conditions for more than 15 years. The metagenomic approach successfully recovered the predominant *Ca.* Scalindua metagenomic MAG, which contained FBTs, but lacked folate biosynthesis genes. In addition, some coexisting bacteria within the anammox consortium possessed THF. Batch experiments with exogenous THF and ^14^C-labeled bicarbonate showed an increase of 43% in the carbon uptake rate. These results support the important role of THF in promoting enzymatic activity during carbon processing without disturbing anammox activity. Future research is needed on other compounds that may enhance anammox activity, accelerate growth rates, and reveal gene expression. Additionally, detailed biochemical and structural studies are essential to fully elucidate the mechanisms of FBTs.

## Citation

Nawarathna, T. N. T. K., Fujii, N., Yamamoto, K., Kuroda, K., Narihiro, T., Ozaki, N., et al. (2025) Metagenomic Insights into *Candidatus* Scalindua in a Long-term Cultivated Marine Anammox Consortium: The Important Role of Tetrahydrofolate-mediated Carbon Fixation. *Microbes Environ ***40**: ME25007.

https://doi.org/10.1264/jsme2.ME25007

## Supplementary Material

Supplementary Material

## Figures and Tables

**Table 1.** Metagenome-assembled genomes obtained from a long-term marine anammox column reactor.

MAG ID	GTDB Taxonomy	Completeness^a^	Contamination^a^	Genome size (bp)	Number of contigs^b^	Abundance (%)
Sca.027	p__*Planctomycetota*;c__*Brocadiae*;o__*Brocadiales*;f__*Scalinduaceae*;g__SCAELEC01	96.6	3.4	5,099,442	11	23.3
Sca.016	p__*Planctomycetota*;c__*Phycisphaerae*;o__*Phycisphaerales*;f__UBA5793;g__PNC21	97.7	1.1	4,497,157	34	19.4
Sca.078	p__*Proteobacteria*;c__*Gammaproteobacteria*;o__QQUJ01;f__QQUJ01;g__QQUJ01	83.1	2.7	3,509,019	12	6.8
Sca.069	p__*Proteobacteria*;c__*Gammaproteobacteria*;o__GCA-2729495;f__GCA-2729495;g__GCA-002729875	86.8	1.5	3,415,451	10	2.6
Sca.039	p__*Myxococcota*;c__*Polyangia*;o__*Haliangiales*;f__*Haliangiaceae*	94.5	5.3	10,004,283	41	2.4
Sca.042	p__*Bacteroidota*;c__*Ignavibacteria*;o__*Ignavibacteriales*;f__*Melioribacteraceae*;g__IGN16	94.1	2.0	4,524,917	25	2.1
Sca.075	p__*Proteobacteria*;c__*Gammaproteobacteria*;o__*Xanthomonadales*;f__*Wenzhouxiangellaceae*;g__*Wenzhouxiangella*	83.6	2.0	2,763,812	14	2.0
Sca.051	p__*Planctomycetota*;c__B15-G4;o__B15-G4;f__JADJEK01	92.5	1.1	7,268,394	36	1.8
Sca.011	p__*Proteobacteria*;c__*Gammaproteobacteria*;o__UBA5335;f__UBA5335;g__*Macondimonas*	98.5	1.2	2,642,206	2	1.8
Sca.002	p__*Actinobacteriota*;c__*Acidimicrobiia*;o__UBA5794;f__UBA5794;g__UBA5794	100	1.3	2,648,439	13	1.7
Sca.012	p__*Bacteroidota*;c__*Bacteroidia*;o__*Flavobacteriales*;f__*Flavobacteriaceae*;g__*Altibacter*	98.5	0	3,109,375	2	1.6
Sca.060	p__*Chloroflexota*;c__*Anaerolineae*;o__*Anaerolineales*;f__UBA11579;g__CFX15	90.9	4.6	4,962,996	5	1.0
Sca.045	p__*Planctomycetota*;c__*Planctomycetia*;o__*Pirellulales*;f__*Pirellulaceae*	93.7	1.1	7,549,545	42	1.0
Sca.028	p__*Planctomycetota*;c__UBA1135;o__UBA2386;f__GCA-002748355	96.6	0.1	5,900,917	20	1.0
Sca.047	p__*Proteobacteria*;c__*Gammaproteobacteria*;o__*Pseudomonadales*;f__HTCC2089;g__SZUA-309	93.5	2.2	4,969,807	15	0.9
Sca.019	p__*Proteobacteria*;c__*Alphaproteobacteria*;o__*Rhodobacterales*;f__*Rhodobacteraceae*;g__*Roseovarius*	97.3	2.8	3,730,243	8	0.9
Sca.017	p__*Spirochaetota*;c__*Leptospirae*;o__*Leptospirales*	97.7	0	3,888,891	12	0.9
Sca.048	p__*Proteobacteria*;c__*Gammaproteobacteria*;o__*Xanthomonadales*;f__*Marinicellaceae*;g__*Marinicella*	93.5	1.5	2,935,724	5	0.9
Sca.074	p__*Myxococcota*_A;c__UBA796;o__UBA796;f__GCA-2862545	83.9	3.9	10,044,473	117	0.7
Sca.040	p__*Planctomycetota*;c__UBA8742;o__SKZP01	94.3	5.7	5,934,693	10	0.7
Sca.044	p__*Proteobacteria*;c__*Alphaproteobacteria*;o__SMXQ01;f__SMXQ01	93.9	2.6	3,077,441	9	0.6
Sca.006	p__*Actinobacteriota*;c__*Actinomycetia*;o__*Mycobacteriales*;f__*Mycobacteriaceae*;g__*Mycobacterium*	98.9	0.6	5,467,850	23	0.6
Sca.082	p__*Chloroflexota*;c__*Anaerolineae*;o__4572-78;f__J111;g__J095	78.5	3.6	7,059,453	58	0.6
Sca.026	p__*Verrucomicrobiota*;c__*Verrucomicrobiae*;o__*Opitutales*;f__UBA2995;g__UBA2995	96.6	4.7	6,670,232	36	0.5
Sca.054	p__*Actinobacteriota*;c__*Acidimicrobiia*;o__*Acidimicrobiales*;f__*Microtrichaceae*	92.3	0.9	4,078,026	21	0.5

^a^ A quality assessment of the assembled genome was conducted using CheckM version 1.2.0. ^b^ Contigs and 16S rRNA from the assembled genome were evaluated using PROKKA version 1.1.3. All MAGs represent >70% completeness, <6% contamination, and >0.5% abundance.
