# Supplementary material for: Metagenomic Insights into Candidatus Scalindua in a Long-term Cultivated Marine Anammox Consortium: The Important Role of Tetrahydrofolate-mediated Carbon Fixation
Source: Microbes Environ. 2025 Jun 17;40(2):ME25007. doi: 10.1264/jsme2.ME25007 (PMC12213060; doi:10.1264/jsme2.ME25007)
Supplement: Supplementary file 1 — Supplementary Material [file 40_25007_s1.pdf]

Supplementary Materials

Metagenomic Insights into *Candidatus* Scalindua in a Long-term Cultivated Marine Anammox Consortium: The Important Role of Tetrahydrofolate-mediated Carbon Fixation

T.N.T.K. Nawarathna, Naoki Fujii, Kohei Yamamoto, Kyohei Kuroda, Takashi Narihiro, Noriatsu Ozaki, Akiyoshi Ohashi, Tomonori Kindaichi

The supplementary material contains 3 figures and 4 tables.

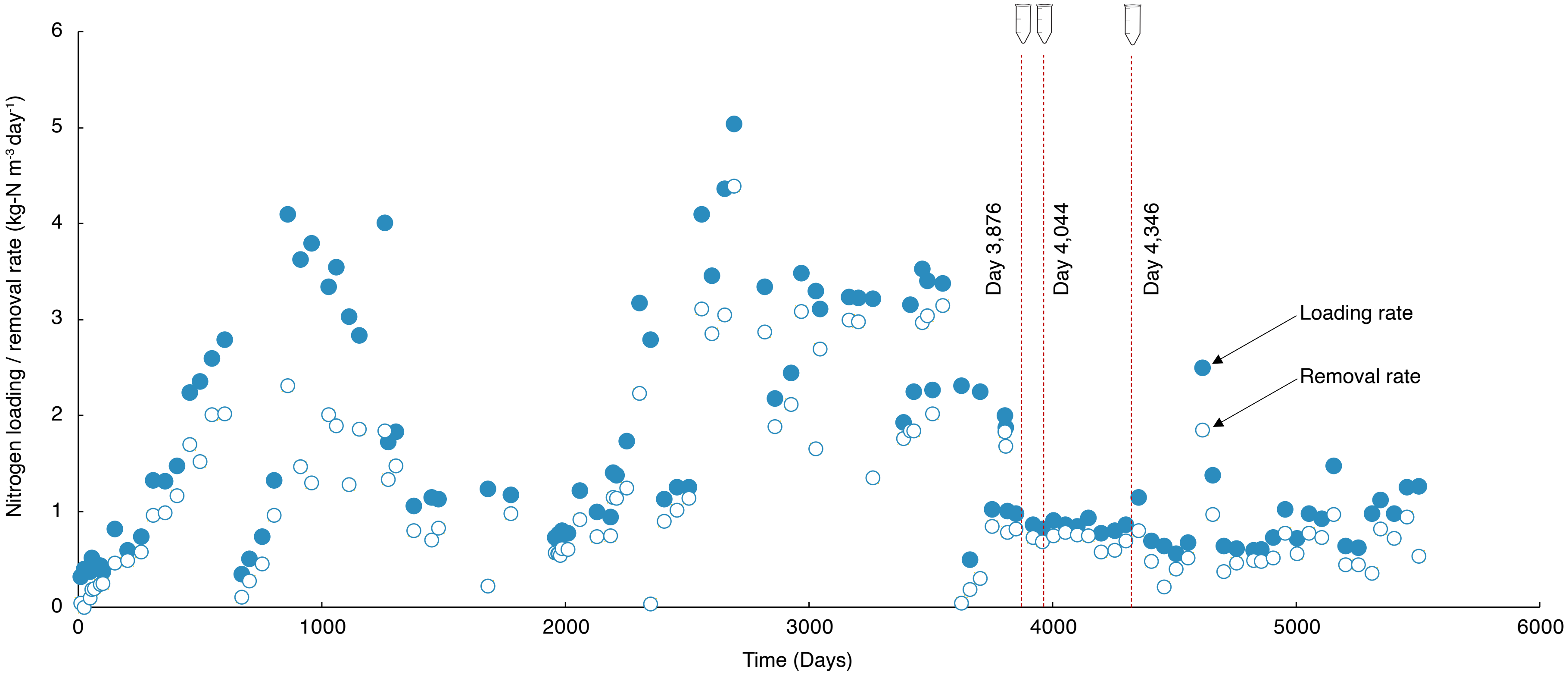

Fig. S1. Nitrogen removal performance of the up-flow column reactor for *Candidatus* Scalindua. Filled and open circles represent nitrogen loading and removal rates, respectively. The biomass samples were collected on days 3,876, 4,044, and 4,346, respectively.

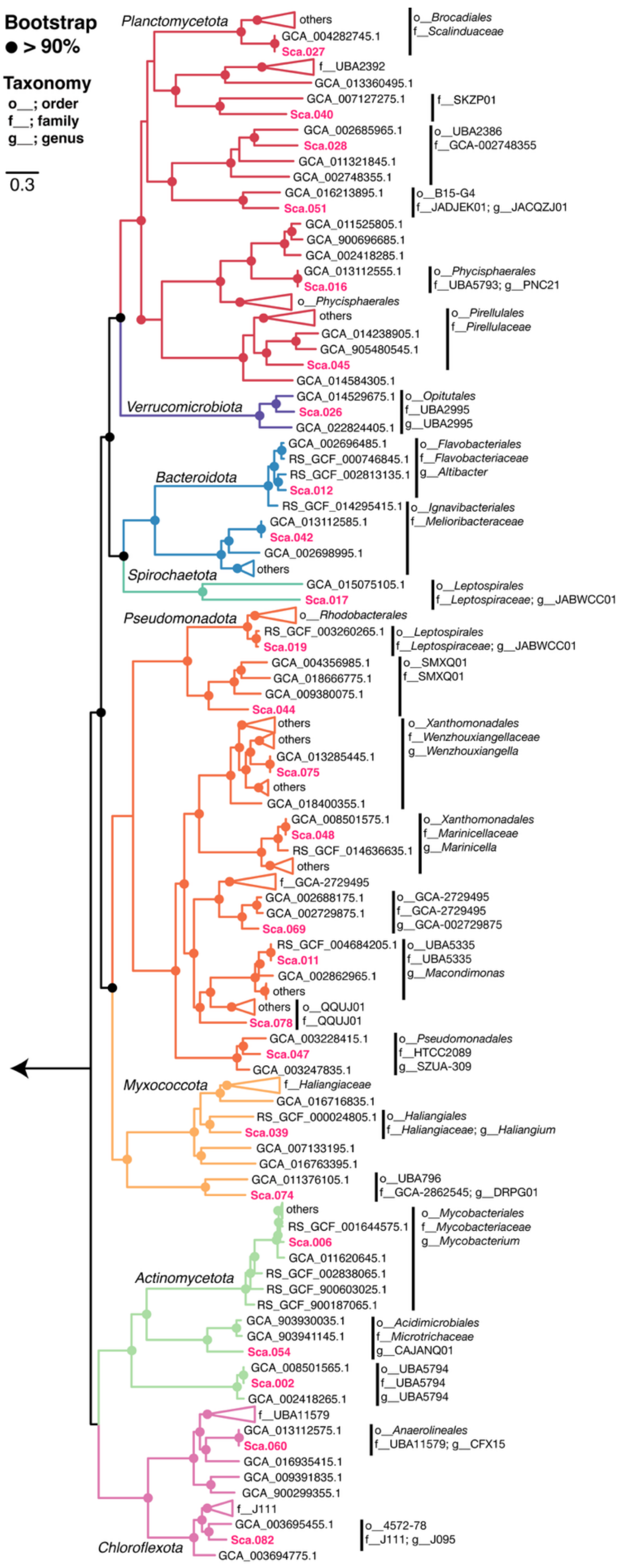

Fig. S2. Phylogenetic tree of 25 high-quality bins (pink) based on the concatenated phylogenetic marker genes from GTDB-Tk v2.2.6 (R207). Circles at the nodes represent bootstrap values >90% obtained from 1,000 re-samplings.

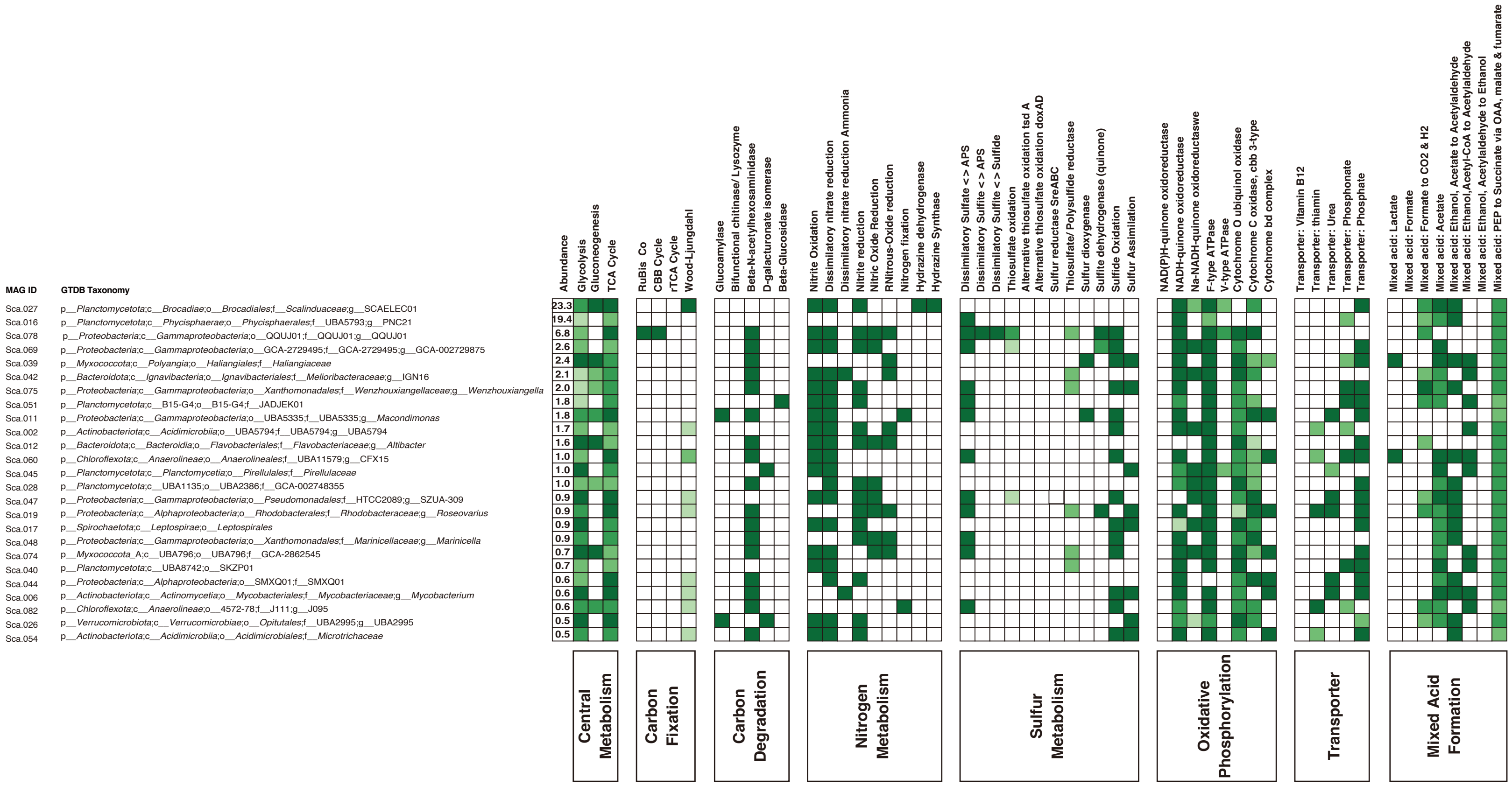

Fig. S3. Heatmap representing metabolic pathway completeness, determined by the presence or absence of genes using KEGG decoder with KEGG annotation. Colors indicate the presence of full or partial genes associated with metabolic pathways.

Table S1. Batch experiment conditions.

**Experiments***	**THF (mg L^{-1})**
Control	0
THF	2
Pasteurized	0

*All experiments were conducted at 28°C for 24 h with 0.35 mg-protein $vial^{-1}$ and 370 kBq of [^{14}C] bicarbonate $vial^{-1}$ in five replicates.

*The synthetic marine medium, containing 70 mg-N L^{-1} of NH_4^+ and NO_2^-, 500 mg L^{-1} of $KHCO_3$, and 3.5% of salinity (SEALIFE) was used for all experiments.

Table S2. Summary of the metagenomic Hiseq X data used in this study.

Platform	Sample	Total reads bases (bp)	Total reads	GC (%)	Q20(%)
HiseqX	HosoScaH190417	44,801,460,514	296,698,414	49.8	97.01
HiseqX	HosoScaH190806	36,472,443,058	241,539,358	55.8	96.73
HiseqX	HosoScaH200603	35,597,576,238	235,745,538	51.5	97.06

Table S3. Summary of the metagenomic PacBio data used in this study.

Platform	Sample	Sub reads bases (bp)	Subreads	Subread N50	Average read length
PacBio	HosoScaH190417	9,670,779,716	1,193,479	11,314	8,103
PacBio	HosoScaH190806	8,005,069,770	1,118,108	10,607	7,159
PacBio	HosoScaH200603	9,000,223,118	1,273,618	10,461	7,066

Table S4. Comparison of folate-related genes and transporters in published *Candidatus* Scalindua MAGs.

MAG ID	Assembly name	Organism name	Assembly level	Completeness	Contamination	folA*	folC*	FBT*
GCA_031316235.1	ASM3131623v1	*Candidatus* Scalindua sp.	Contig	97.73	4.55	×	○	○
GCA_022181045.1	ASM2218104v1	*Candidatus* Scalindua sp.	Scaffold	96.59	3.41	×	○	○
GCA_008501815.1	ASM850181v1	*Candidatus* Scalindua sp.	Contig	96.59	3.41	×	○	○
GCA_004282745.1	ASM428274v1	*Candidatus* Scalindua sp. SCAELEC01	Scaffold	96.59	3.41	×	○	○
GCA_964194795.1	07C-BC-St03_bin.21	uncultured *Candidatus* Scalindua sp.	Scaffold	96.58	5.13	×	○	×
GCF_002443295.1	ASM244329v1	*Candidatus* Scalindua japonica	Contig	95.45	3.41	×	○	○
GCA_033023575.1	ASM3302357v1	*Candidatus* Scalindua sp.	Scaffold	95.45	2.27	×	○	×
GCA_030748215.1	ASM3074821v1	*Candidatus* Scalindua sp.	Contig	95.45	2.27	×	○	×
GCA_017368835.1	ASM1736883v1	*Candidatus* Scalindua sediminis	Scaffold	95.45	3.41	×	○	×
GCA_008636105.1	ASM863610v1	*Candidatus* Scalindua sediminis	Scaffold	95.45	4.55	×	×	×
GCA_964344685.1	ERP019367_co2_59	uncultured *Candidatus* Scalindua sp.	Scaffold	94.32	2.27	×	○	×
GCA_033023715.1	ASM3302371v1	*Candidatus* Scalindua sp.	Scaffold	94.32	2.27	×	○	×
GCA_031316155.1	ASM3131615v1	*Candidatus* Scalindua sp.	Contig	94.32	3.41	×	○	×
GCA_019911885.1	ASM1991188v1	*Candidatus* Scalindua rubra	Contig	93.87	2.27	×	○	×
GCA_021776915.1	ASM2177691v1	*Candidatus* Scalindua sp.	Contig	93.18	2.27	×	○	○
GCA_018670825.1	ASM1867082v1	*Candidatus* Scalindua sp.	Contig	93.18	4.61	×	○	×
GCA_018263435.1	ASM1826343v1	*Candidatus* Scalindua arabica	Contig	93.18	2.27	×	○	×
GCF_000786775.1	ASM78677v1	*Candidatus* Scalindua brodae	Contig	92.74	2.27	×	○	×
GCA_001723765.1	ASM172376v1	*Candidatus* Scalindua rubra	Contig	92.52	5.11	×	○	×
GCA_964437425.1	SRP167671_co2_43	uncultured *Candidatus* Scalindua sp.	Scaffold	92.05	2.27	×	○	×
GCA_018655105.1	ASM1865510v1	*Candidatus* Scalindua sp.	Contig	92.05	4.61	×	○	×
GCA_018662645.1	ASM1866264v1	*Candidatus* Scalindua sp.	Contig	90.91	3.48	×	○	×
GCA_002632345.1	ASM263234v1	*Candidatus* Scalindua rubra	Contig	90.46	2.27	×	○	×
GCA_964584595.1	SRP200020_co5_90	uncultured *Candidatus* Scalindua sp.	Scaffold	88.64	3.51	×	○	×
GCA_024654465.1	ASM2465446v1	*Candidatus* Scalindua sp.	Contig	83.65	2.27	×	○	×
GCA_046757745.1	ASM4675774v1	*Candidatus* Scalindua sp.	Contig	83.25	1.24	×	○	×
GCA_018700855.1	ASM1870085v1	*Candidatus* Scalindua sp.	Contig	80.68	4.72	×	×	×
GCA_018675875.1	ASM1867587v1	*Candidatus* Scalindua sp.	Contig	80.68	3.48	×	○	×
GCA_024654085.1	ASM2465408v1	*Candidatus* Scalindua sp.	Contig	80.24	2.79	×	○	×
GCA_036477455.1	ASM3647745v1	*Candidatus* Scalindua sediminis	Contig	72.73	0	×	○	×

*Presence (○) or lack (×) of genes was confirmed using BlastKOALA.
